# Seasonal occurrence of brown marmorated stink bug and its impact in organic and conventional kiwifruit orchards in north‐western China

**DOI:** 10.1002/ps.8812

**Published:** 2025-04-15

**Authors:** Jin‐Ping Zhang, Ju‐Hong Chen, Zhan‐De Liu, Wen‐Jing Li, Maryam Alavi, Xin‐Yue Tian, Shu‐Sen Shi, Feng Zhang, Gonzalo A. Avila

**Affiliations:** ^1^ MARA‐CABI Joint Laboratory for Bio‐safety Institute of Plant Protection, Chinese Academy of Agricultural Sciences Beijing China; ^2^ CABI Beijing China; ^3^ College of Horticulture Northwest A&F University Yangling China; ^4^ The New Zealand Institute for Plant and Food Research Limited Auckland New Zealand; ^5^ College of Plant Protection Jilin Agricultural University Changchun China

**Keywords:** *Halyomorpha halys*, phenology, injury level, *Actinidia chinensis*, pest management

## Abstract

**BACKGROUND:**

*Halyomorpha halys*, the brown marmorated stink bug (BMSB), is a significant pest in agricultural and horticulture, with sporadic outbreaks reported in kiwifruit orchards in China, Italy, and Greece. However, information on its seasonal occurrence and impact on kiwifruit remains limited. The present study was conducted to investigate the patterns of seasonal occurrence of BMSB in 2019, 2021 and 2022, as well as their feeding damage in kiwifruit orchards with different management systems (conventional and organic) in Shaanxi Province, China in 2019 and 2021.

**RESULTS:**

BMSB populations were slightly higher in the organic orchard, with two distinctive generations recorded annually and three adult population peaks detected by pheromone traps in both organic and conventional experimental orchards. Overall, the first peak, from overwintering adults, occurred in mid‐May, the second peak in mid‐July, and the largest peak in mid‐ to late‐September. Nymphs started to be detected in late‐May and continued to occur throughout the season. Two peaks of eggs were observed: the first from mid‐May to early‐June and the second from mid‐July to early‐August. Weekly assessments revealed feeding damage in kiwifruit from fruit set to harvest, peaking at 40% in conventional and 59% in organic orchards in October 2019. In 2021, peak damages reached 30% and 60% in conventional and organic orchards, respectively, with no significant differences in overall damage rates between the systems.

**CONCLUSION:**

Our investigation highlights critical periods for utilizing parasitoids in biological control, based on the dynamics of BMSB populations and their associated damage within the kiwifruit orchards evaluated. © 2025 The Author(s). *Pest Management Science* published by John Wiley & Sons Ltd on behalf of Society of Chemical Industry.

## Graphical abstract

Higher populations of brown marmorated stink bug (BMSB) and increased kiwifruit damage were recorded in organic orchards, BMSB population exhibiting a consistent pattern of two generations annually over 3 years. Feeding damage peaked at 59% and 60% in October 2019 and 2021, respectively, with no significant differences in overall damage rates between management systems.

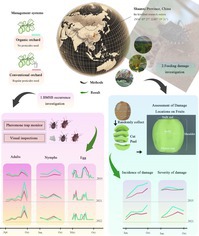

## 1 INTRODUCTION

The brown marmorated stink bug (BMSB), *Halyomorpha halys* Stål (Hemiptera: Pentatomidae) has become a serious agricultural insect pest worldwide. It is a highly polyphagous invasive species with a broad host range of over 170 plants.^1^ Confirmed host plants include many economically important horticultural crops, and ornamentals trees.^2^ Damage is caused by nymphs and adults feeding on flower buds, fruits, or stems when injecting digestive enzymes to feed on the fluids of host plants. This leads to scarred and deformed fruit and leaves and can also cause flower/fruit drop.^3^ In addition, BMSB feeding damage may make fruits and plants more susceptible to secondary fungal infections, resulting in significant economic losses in fruit/crop quality and overall yield, as well as increase costs in pest management practices.^1^ Currently, chemical control is the most widely used tactic for managing BMSB in commercial orchards.^2^ However, extensive use of pesticides creates several adverse effects such as environmental pollution and pesticide residues in food, which have negative effects on human and animal health.^4^, ^5^ Moreover, the intensive use of broad‐spectrum pesticides may also have adverse effects on the coevolved natural enemies that suppress BMSB population in the agroecosystem.^6^ Therefore, more sustainable pest management approaches are urgently needed for BMSB control.

Green kiwifruit, *Actinidia chinensis* var. *deliciosa* ‘Hayward’ is the most common commercial variety worldwide, especially in China,^7^ New Zealand, Italy and Chile.^8^ In recent years, commercial kiwifruit production has been threatened by BMSB. In China, BMSB is a dominant stink bug species, accounting for 92.2% of all the pest stink bugs on kiwifruit^9^ and has been reported as an increasingly damaging species.^10^, ^11^ In Italy, BMSB feeding damage has been reported to cause approximately 30% yield losses in kiwifruit orchards.^12^ In New Zealand, although BMSB is not yet present, it is considered as a major potential threat to the kiwifruit sector.^13^ However, despite the acknowledged BMSB threat to kiwifruit, no specific management recommendations have yet been made to control BMSB in kiwifruit orchards. The spatiotemporal and seasonal distribution patterns varied of BMSB according to habitat.^14^, ^15^ To develop an effective pest management strategy against BMSB for kiwifruit, it is essential to understand the seasonal phenology of BMSB in kiwifruit orchards, during fruit growth periods as well as its impact to kiwifruit. Traps loaded with pheromone lures have been confirmed to be reliable tools to monitor BMSB in different geographical locations with varying population densities throughout the season.16^16^, ^17^ More adult and nymphal BMSB were captured at varying heights compared to near the ground,^18^ this further supported that pheromone traps provided effective decision support tools for pest management decisions.^19^

BMSB feeding time in different crop cultivars were identified as major factors affecting injury expression and severity, and damage characters and levels varied among crops.^2^ Chen *et al*.^10^ demonstrated that both BMSB nymphs and adults caused green and white damage spots under the skin of kiwifruit, and the proportion of each type of damage varied with the fruit development stage and the cultivar. The damage symptoms were not visible from the surface of the fruit after BMSB piercing.^9^ However, localized wilting and necrotic spots were easily observed when the skin of kiwifruit was peeled off. Moreover, when compared with healthy fruit, the BMSB‐damaged ones rapidly became rotten during cold storage.^9^ The losses could be observed during cold storage or shelf stage although the damage occurred during the kiwifruit field growing season.

In the present study, we investigated the patterns of seasonal occurrence of BMSB eggs, nymphs, and adults (female and male), as well as the feeding damage caused by BMSB in kiwifruit orchards with different management systems (organic *versus* conventional). BMSB seasonal occurrence was assessed during the 2019, 2021 and 2022 seasons, while feeding damage in kiwifruit was assessed during the 2019 and 2021. The implications of the results of these study in the implementation of biological control practices against BMSB in kiwifruit orchards are discussed.

## 2 MATERIALS AND METHODS

### 2.1 Experimental condition

The study was conducted in a conventional (i.e., pesticides sprayed) and an organic (i.e., no spray of pesticides) kiwifruit orchard in Mei County, Shaanxi Province, China. The organic orchard was located at the kiwifruit research station of the Northwest Agricultural and Forestry University (34° 07′ 27″ N; 107° 59′ 31″ E), where no pesticide spray was used at all. Our field trials were conducted on *Actinidia chinensis* var. *deliciosa* ‘Hayward’ cultivar, occupying 12 800 m^2^ (80 m × 160 m). The conventional orchard (34°08′06″ N; 107°59′24″ E) [In 2019, two cyhalothrin sprays (April and June) and one of bifenthrin (May) were applied. In 2021, one application each of Cyhalothrin (April), Imidacloprid (April), Bifenthrin (May), and Pyradaben (July) were applied.] used in this study, which was located 2000 m away from the organic orchard, and has a land size of 1700 m^2^ (10 m × 170 m).

### 2.2 Occurrence and population dynamics of BMSB in kiwifruit orchards

Three pyramid traps (1.22 m height, AgBio Inc., Westminster, CO, USA) loaded with aggregation pheromone lures (PHEROCON BMSB DUAL™ ‘High Load’, TRÉCÉ, Inc., Adair, IA, USA) were deployed in each orchard, to monitor the presence of BMSB nymphs and adults and assess population dynamics from 18 April to 26 November 2019, 2 March to 16 October 2021, and 12 March to 18 October 2022. In both conventional and organic orchards, one trap was set up in the middle of the experimental block, and two additional traps were set up at the extent line in both directions, each with approximately 100 m from the middle one. Pheromone lures were changed every 3 months. All traps were checked weekly for BMSB nymphs and adults, and all insects found in traps were recovered and taken back to the laboratory for numbers and sex recording. Visual inspections were also conducted to complement data obtained from pheromone trapping, especially to acquire the egg occurrence data. In each experimental orchard (conventional or organic) 20 kiwifruit vines were randomly selected and visually inspected weekly for BMSB eggs, nymphs and adults, from flowering to harvest. The observation interval was the same as the pheromone trap check interval in both 2019 and 2021. Each tree was observed from four directions (east, south, west and north) and at least 20 leaves and ten fruits were inspected for the presence of BMSB. The number of eggs, nymphs, and adults of BMSB were recorded during each visual inspection.

### 2.3 Evaluation of BMSB feeding damage in kiwifruit orchards

**Figure 1:**
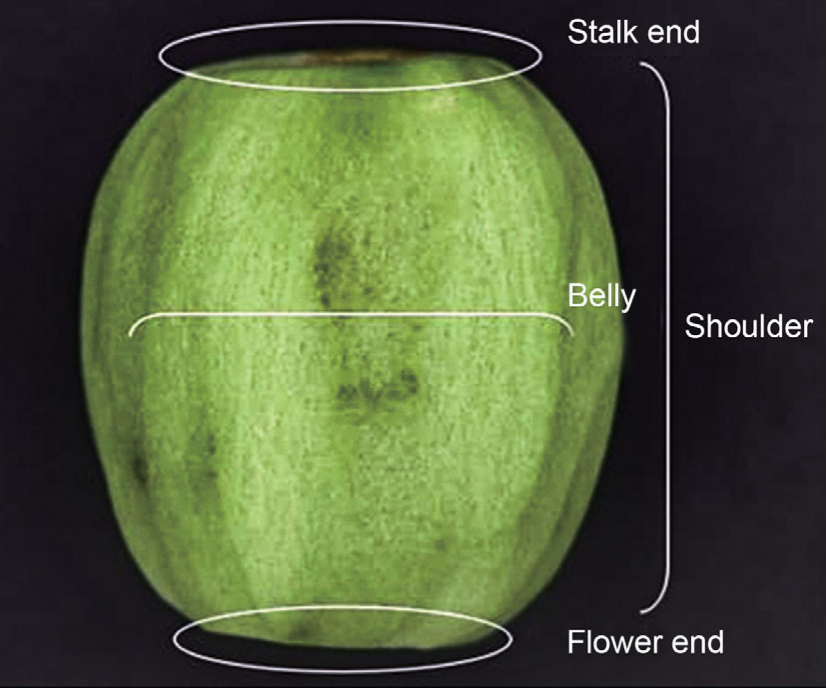
Position within kiwifruit used to assess location of *Halyomorpha halys* feeding damage.

Assessment of incidence (percentage of kiwifruit damaged) and severity (number of damage spots per damaged kiwifruit) of feeding damage in kiwifruit, caused by naturally occurring BMSB, was conducted in both organic and conventional orchards. A sample of 30 kiwifruits was randomly collected from each orchard in weekly intervals from the beginning of fruit development until harvest (i.e., June–October) in both 2019 and 2021. All fruits collected were brought back into the laboratory, where fruit skin was peeled off and cut into four even pieces and inspected for BMSB feeding damage assessment. Total number of damage spots observed were recorded to determine the damage position within the fruit (i.e., shoulder, belly, stalk end and flower end, Fig. 1), the incidence (percentage of kiwifruit damaged) and severity of damage (mean number of spots per damaged kiwifruit). Damage spots were assessed based on feeding damage characteristics reported in kiwifruit by Chen *et al*.^13^ and Lara *et al*.^20^

### 2.4 Statistical analysis

Seasonal occurrence of BMSB data recorded by pheromone trapping were analysed at each life stage [average nymph (all nymphal stages combined) and adult]. Generalized linear mixed model (GLMM) was used on the counts of adults and nymphs with Poisson distribution at log scale. Management system (i.e., organic and conventional), experimental months and their interactions were used as fixed effects and trap number at each site was used as the block effect. A chi‐squared test was used for significance of the fixed effects. Monitoring activities via visual inspections during all experimental seasons were not effective to produce robust data, so no formal statistical analysis could be performed. Therefore, count data for visual inspections are presented as raw data only to allow visualization. Feeding damage data was analysed using GLMMs. The incidence of fruit damage was analysed using a GLMM with binomial errors (link = logit), where damage = 1 indicates binomial success and damage = 0 as binomial failure. The severity of damage was analysed using a GLMM with Poisson errors (link = log). For both models, management system, experimental months and their interactions were as fixed effects and collection week was the block effect.

A chi‐squared test was employed to test the statistical significance of the fixed effects in both models. Damage spots depending on the damage location (shoulder, belly, stalk end or flower end) are presented as raw percentages to allow visualization.

Owing to the lack of spatial replication, we note that although the management system was a fixed effect, its statistical significance cannot be generalized but is seen as a preliminary indication only on the potential effect of management systems on BMSB occurrence and feeding damage. All computations and graphics were performed using R in R Studio platform, version 1.1.422, using lme4, predict means, and ggplot2 packages.

## 3 RESULTS

### 3.1 Population occurrence and dynamics of BMSB in kiwifruit

#### 3.1.1 Pheromone trapping

Both nymphs and adults BMSB were captured in pheromone traps. A total of 1209, 1245, and 1279 nymphs were captured during the whole monitoring season in 2019, 2021 and 2022, respectively. Captures of BMSB adults were two‐fold higher than nymphs in 2019 and 2021, and three‐fold in 2022 (Table 1).

**Table 1:** Total number of *Halyomorpha halys*, brown marmorated stink bug (BMSB), nymphs and adults recovered from pheromone traps deployed in organic and conventional ‘Hayward’ kiwifruit orchards in 2019, 2021, and 2022 experimental season

Year	Management system	Number of BMSB adults	Number of BMSB nymphs
2019	Conventional	848	331
	Organic	1591	878
2021	Conventional	1037	554
	Organic	1868	691
2022	Conventional	1538	665
	Organic	2036	614

**Figure 2:**
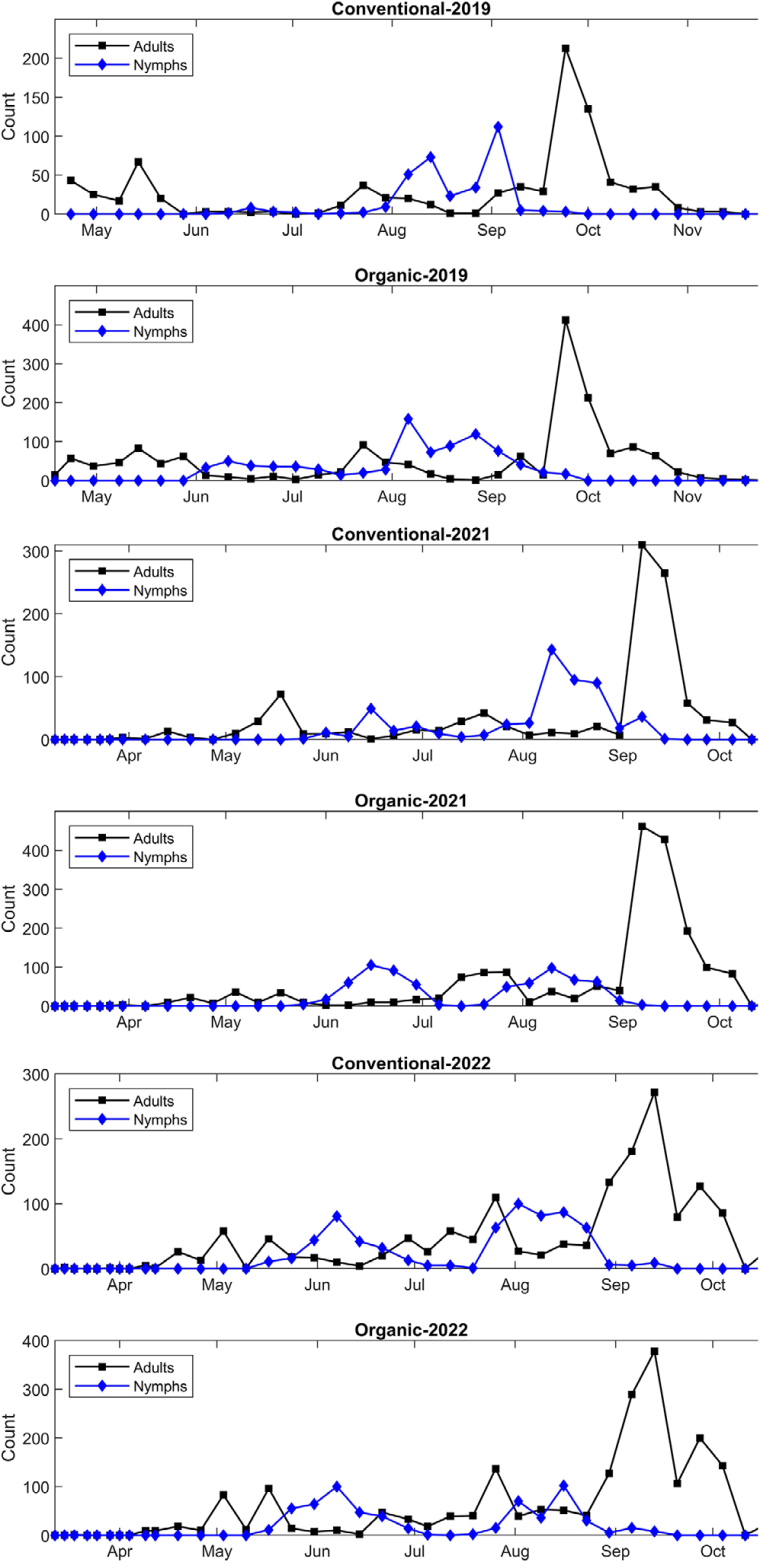
Relative abundance (raw data) of *Halyomorpha halys* adults recovered monthly from pheromone traps deployed in experimental conventional and organic ‘Hayward’ kiwifruit orchards in 2019, 2021, and 2022.

BMSB population dynamics in both orchards showed a similar pattern in all three experimental years. BMSB adults were mostly captured from late April until late‐October, and three peaks were observed during each sampling year in both experimental orchards (Fig. 2). Nymphs were captured mostly from early June to late August, and each year two peaks were observed in both experimental orchards.

A first peak in BMSB adults was observed in both orchards from mid‐ to late‐May in 2019 and 2021, and from early‐ to mid‐May in 2022 (Fig. 2). This first peak corresponds to overwintering adults from the previous year that started to resume activity. A second peak of BMSB adults (first BMSB generation) was observed in 2019 from late‐July to early‐August, and from mid‐ to late‐July in 2021 and 2022 (Fig. 2). The third and highest peak (*P* < 0.01), which corresponds to the second BMSB generation, was observed around late‐September in 2019, and early‐ to mid‐September in 2021 and 2022 (Fig. 2). A first peak in BMSB nymphs was observed in both orchards around early‐ to mid‐June in 2019 and 2021, and from late‐May to early‐June in 2022 (Fig. 2). The second and highest (*P* < 0.01) peak of BMSB nymphs was observed from early‐August to early‐September in 2019, and from early‐ to late‐August in 2021 and 2022 (Fig. 2).

**Figure 3:**
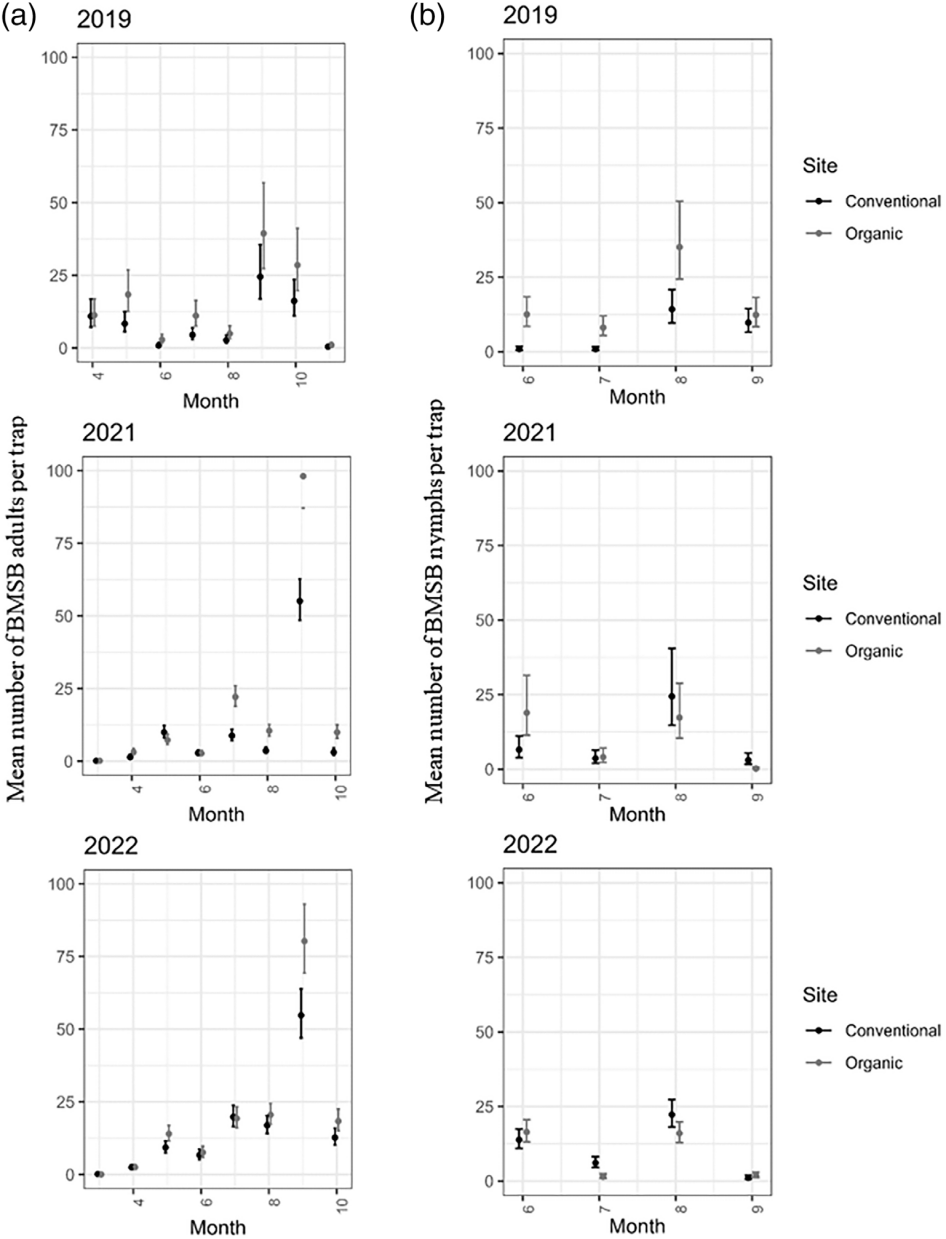
Estimated mean number of *Halyomorpha halys*, brown marmorated stink bug (BMSB), (a) adults and (b) nymphs (with 95% confidence intervals) recovered pheromone traps deployed in organic and conventional ‘Hayward’ kiwifruit orchards deployed in 2019, 2021, and 2022. The circles are the back‐transformed predicted means by a generalized linear mixed model (GLMM) and the vertical lines show back‐transformed confidence intervals. Non‐overlapping confidence intervals within orchard sites suggest evidence of statistically significant difference.

In 2019, the mean number of adults per trap in the organic orchard was significantly higher (*P* < 0.01) than the conventional orchard in May and July (Fig. 3(a)). In 2021, mean number of adults per trap in the organic orchard was significantly higher (*P* < 0.01) than the conventional orchard from July to October, however in 2022, this was observed only in September (Fig. 3(a)).

The mean number of nymphs recovered per trap in the organic orchard during 2019 was significantly higher (*P* < 0.01) than the conventional orchard from June to August (Fig. 3(b)). In 2021, mean number of nymphs per trap in the organic orchard was significantly higher (*P* < 0.01) than the conventional orchard in June only, however in 2022, the mean number of nymphs in the organic orchard were not higher than the conventional orchard (Fig. 3(b)).

#### 3.1.2 Visual inspections

**Figure 4:**
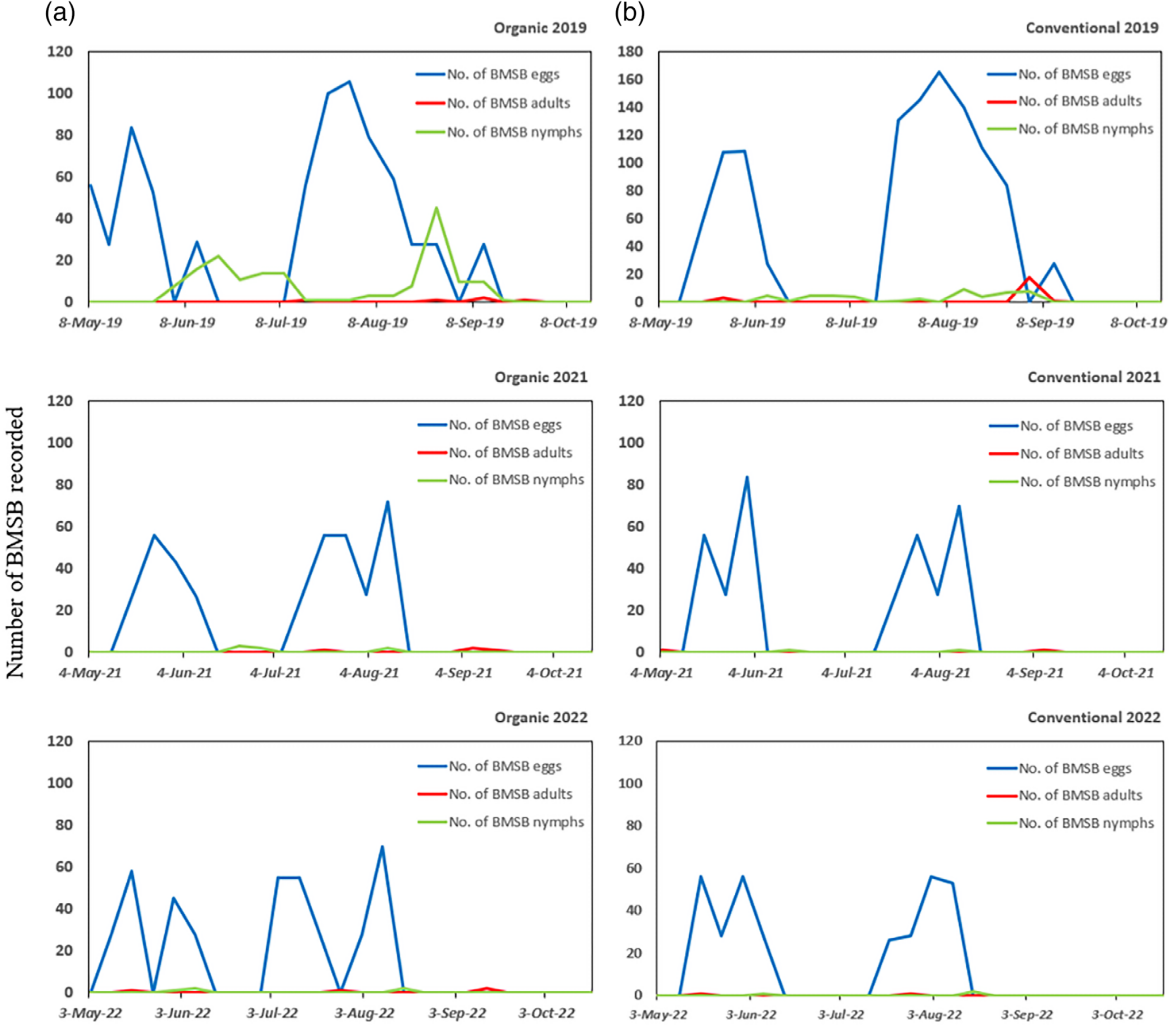
Relative abundance (raw data) of *Halyomorpha halys*, brown marmorated stink bug (BMSB), eggs, egg masses, nymphs and adults recorded in (a) organic and (b) conventional ‘Hayward’ kiwifruit orchards during weekly visual inspections conducted in 2019, 2021, and 2022.

A total of 1841 BMSB eggs from 68 masses (2019), 744 eggs from 29 masses (2021), and 726 eggs from 26 masses (2022) were recorded from visual inspections in kiwifruit. Total number of nymphs and adults recorded during visual inspections was quite low in all years, being 2019 when more nymphs (219) and adults (27) were observed (Table 2 and Fig. 4).

**Table 2:** Total number of *Halyomorpha halys*, brown marmorated stink bug (BMSB), eggs, egg masses, nymphs and adults recorded during visual inspections conducted in organic and conventional ‘Hayward’ kiwifruit orchards in 2019, 2021, and 2022 experimental seasons

Year	Management system	Number of BMSB eggs	Number of BMSB egg masses	Number of BMSB nymphs	Number of BMSB adults
2019	Conventional	1107	40	51	22
	Organic	734	28	168	5
2021	Conventional	350	13	2	2
	Organic	394	16	7	4
2022	Conventional	331	12	3	2
	Organic	395	14	5	4

Two peaks of BMSB eggs were found during visual observations in both organic and conventional orchard. The first peak was observed from mid‐May to early‐June and the second peak between mid‐July to early‐August, which was consistent in both experimental orchards in all experimental years (Fig. 4(a),(b)).

Overall, visual inspections were effective to gather information on the time within the experimental season when BMSB eggs are present in the field and when peaks occur, which was useful to visualize when BMSB generations start. However, this method was not effective to monitor abundance of nymph and adult BMSB in kiwifruit vines.

### 3.2 Incidence and severity of BMSB feeding damage caused in kiwifruit

**Figure 5:**
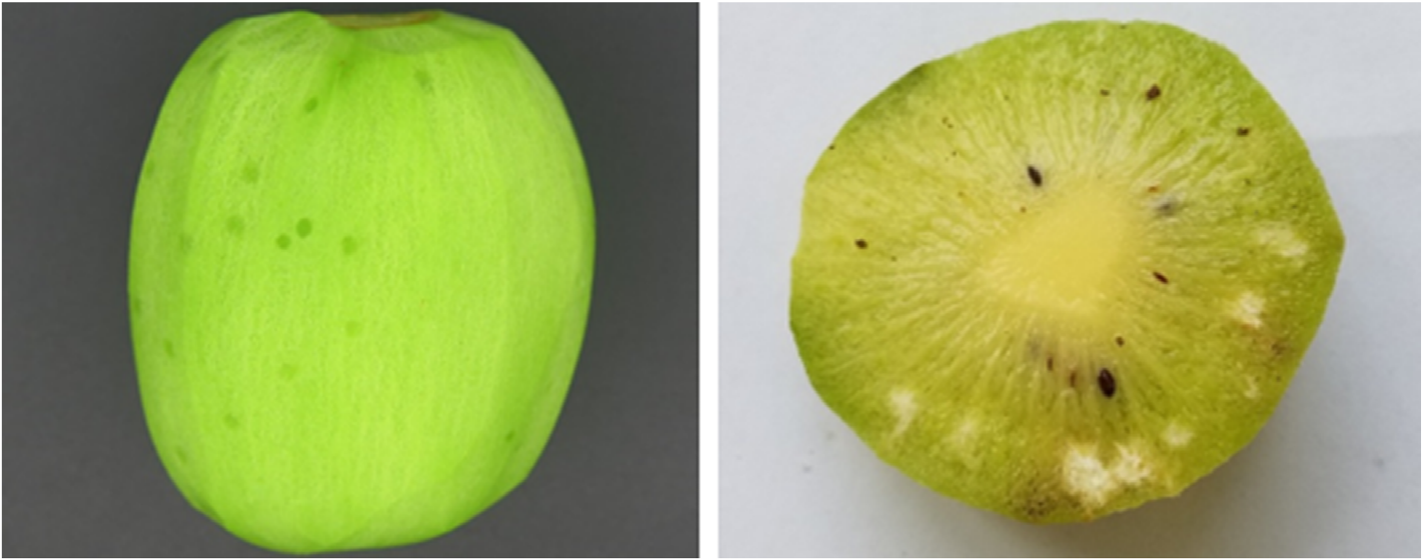
Types of *Halyomorpha halys*, brown marmorated stink bug (BMSB), feeding spots observed in ‘Hayward’ kiwifruit within kiwifruit.

**Figure 6:**
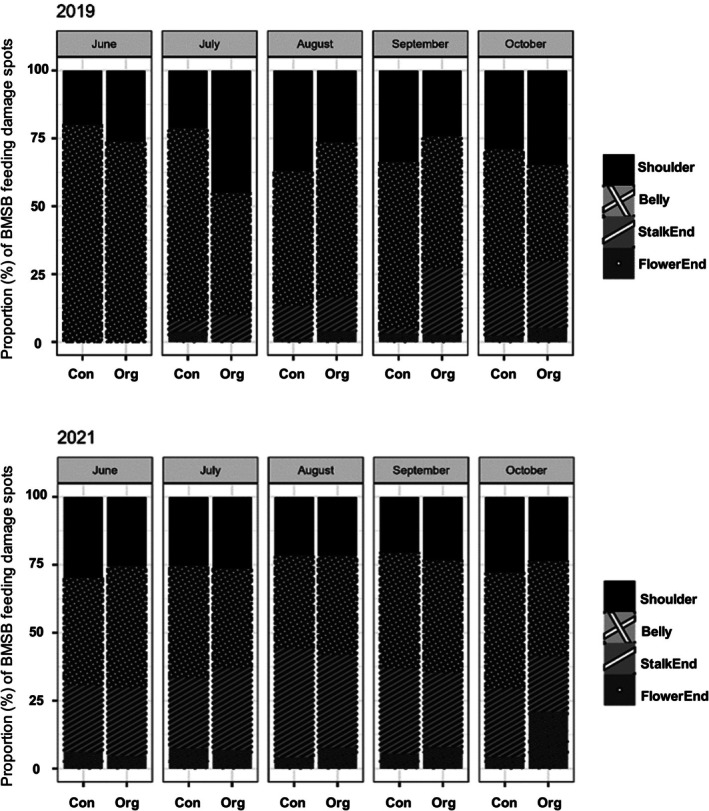
Relative severity of *Halyomorpha halys*, brown marmorated stink bug (BMSB), feeding damage (pooled raw data) observed in ‘Hayward’ kiwifruit during damage assessments conducted in 2019 and 2021. Arithmetic means are shown without standard errors to allow visualization, as the underlying distribution are not normal.

BMSB feeding damage spots (Fig. 5) were found on all four examined positions within the fruit (i.e., shoulder, belly, stalk end and flower end) in both organic and conventional orchards (Fig. 6). In 2019, in both organic and conventional orchards, the majority of BMSB feeding damage spots were found in the belly and shoulder of the fruit with an average of 50% and 32% of damage spots, respectively, over the two orchards (Fig. 6). During 2021, BMSB feeding damage spots were mostly observed in belly and stalk end of the fruit in both conventional and organic orchards. Average damage spots observed over the two orchards was 38% and 23% in belly and stalk end, respectively (Fig. 6).

**Figure 7:**
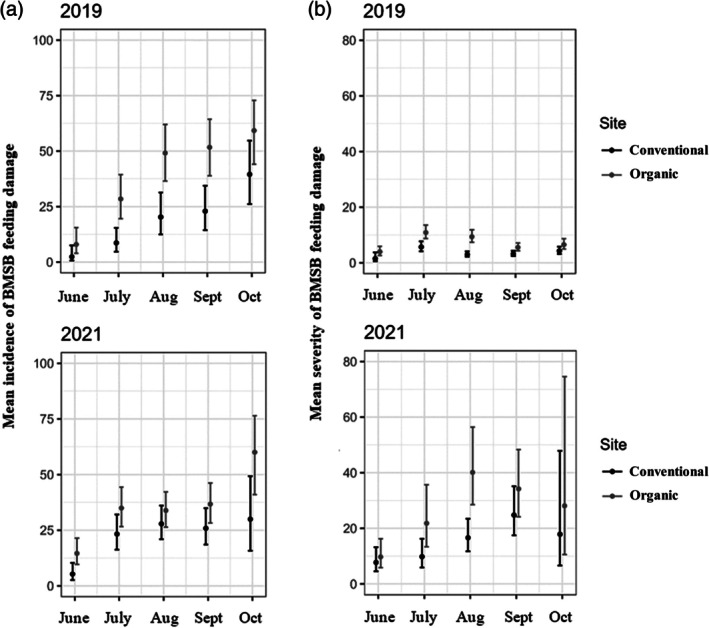
Estimated mean (a) incidence and (b) severity of *Halyomorpha halys*, brown marmorated stink bug (BMSB), feeding damage (with 95% confidence intervals) observed in organic and conventional ‘Hayward’ kiwifruit orchards during 2019 and 2021. The circles are the back‐transformed predicted means by a generalized linear mixed model (GLMM) and the vertical lines show back‐transformed confidence intervals. Non‐overlapping confidence intervals within orchard sites suggest evidence of statistically significant difference.

Overall incidence (i.e., percentage of kiwifruits with confirmed BMSB feeding damage) of fruit damage observed in kiwifruit across the two experimental orchards was consistent between the 2 years of assessments, which was 28% and 26% in 2019 and 2021, respectively. The incidence of fruit damage observed during the 2019 and 2021 sampling periods differed significantly (*P* < 0.01) within the sampling season in both experimental kiwifruit orchards (Fig. 7(a)). In 2019, incidence increased consistently during the fruit growing season and differed significantly (*P* < 0.01) within each orchard, with a peak in mean incidence of 40% and 59% recorded in October in conventional and organic orchards, respectively (Fig. 7(a)). However, overall incidence of fruit damage between conventional orchard (18%) and organic orchard (38%) did not differ significantly. During 2021 fruit sampling season, incidence increased consistently from June to October in both experimental orchards, reaching in October the highest mean incidence of fruit damage in conventional orchard (30%) and organic orchard (60%), respectively (Fig. 7(a)). Overall incidence of fruit damage was 20%, and 31% in the conventional and organic orchards, respectively, which did not differ significantly (Fig. 7(a)).

Similar to incidence of BMSB feeding damage, severity of fruit damage (i.e., number of spots per damaged kiwifruit) observed in kiwifruit was consistent between the 2 years of assessments. A mean number of seven and six damage spots per damaged fruit was observed 2019 and 2021, respectively. Severity of BMSB feeding damage differed significantly (*P* < 0.01) between months in 2019 and 2021 sampling seasons in both organic and conventional kiwifruit orchards (Fig. 7(b)). Severity of damage increased slightly during 2019 fruit growing season in both experimental orchards, with mean severity slightly peaking up in July in both conventional orchard (6 spots/fruit) and organic orchard (11 spots/fruit). Severity of fruit damage differed significantly (*P* < 0.01) between experimental orchards in July and August only (Fig. 7(b)). In 2021, severity peaked slightly in August in the organic orchard (40 spots/fruit) and September in the conventional orchard (25 spots/fruit), respectively. Severity of fruit damage differed significantly (*P* < 0.01) between experimental orchards in August only (Fig. 7(b)).

## 4 DISCUSSION AND CONCLUSION

This study provided first‐hand information on the phenology of BMSB on kiwifruit orchards in north‐western China. Overwintering adults were already present on kiwifruit plants at the end of March when pheromone traps were first checked in 2021. Pheromone trapping revealed three peaks in the adult population in all monitoring years, first for overwintering adults from previous season and then, two more which represented the actual BMSB generations during the fruit growing season. Moreover, there were two peaks observed for BMSB egg masses by visual inspections. The presence of BMSB nymphs was sustained from the beginning of June to end September, where most nymphs were captured between August and September due to overlapping generations. Our results thus confirmed the presence of two BMSB generations per year occurring in the surveyed organic and conventional kiwifruit orchards. This is inconsistent with one generation BMSB occurrence in kiwifruit orchard in Zhouzhi County, Shaanxi Province.^21^ However, most research reported two generations of BMSB per year, such as in south of the Alps, Italy,^22^ mid‐Atlantic region of the United States,^1^, ^23^ Beijing northern China,^24^ and western Slovenia,^25^ although BMSB is strictly univoltine in Switzerland and in central Slovenia.^17^, ^26^

The egg masses were found earlier in organic orchard than conventional orchard. Two occurrence peaks for BMSB eggs were observed during visual inspections in both organic and conventional orchards. The first peak was observed from early May to early June, and the second peak from early July to middle August, across both years. This information is instrumental for the development of pest management programmes against BMSB in the kiwifruit orchards, and in particular for biological control practices. For instance, a time starting 1 week before the appearance of egg masses and the emergence of nymphs could be the optimum time for conducting augmentative releases of egg parasitoids to control BMSB. As far as biological control is considered, releases of biological control agents must be timed to coincide with specific life stages of the pest, particularly if not all life stages are equally vulnerable to the control measures.^27^ BMSB egg parasitoids, such as *Trissolcus japonicus* (Hymenoptera: Scelionidae) and *Anastatus japonicus* (Hymenoptera: Eupelmidae), were confirmed to be the two dominant egg parasitoids of BMSB in northern China.^6^ In addition, the biological control potential of *T. japonicus* and *Anastatus japonicus* has been tested by singly or combined releases in exclusion cages on kiwifruit vines.^28^ Also, *T. japonicus* and *Trissolcus cultratus* have been confirmed as the most abundant parasitoids in kiwifruit orchard in Shaanxi Province, China.^29^ Thus, based on the phenology observed in the study orchards and observations made by Avila *et al*.^29^ on parasitoids abundance and diversity in kiwifruit orchard in Shaanxi Province, China (same orchards used in our study), we suggest conducting augmentative releases of either *T. japonicus* or *T. cultratus* in early May (i.e., start of first BMSB generation), to attempt suppression of the first BMSB generation. However, additional research is needed to accurately determine an optimum release frequency and numbers of parasitoids to release in each release event. A second critical time to conduct releases would be by mid‐ to end‐July and early August, when the second generation of BMSB egg masses occur in the field.

The biggest number of BMSB adults were observed in September for all 3 years, thereafter, the number of adults gradually decreased to zero in November. It is known that BMSB adults aggregate in human‐made structures to overwinter inside protected environments.^30^ Since the average fecundity of overwintering females is higher than the summer generations,^22^ controlling the overwintering BMSB is likely another effective approach to reduce BMSB population in the following year. Initial research efforts have been made in the development of artificial overwintering traps to catch BMSB populations during the migration period in autumn.^31^ Recently several new types of artificial traps were developed and efficiency tested for catching BMSB adults when they aggregate to overwintering sites.^32^ In addition, the tachinid fly *Pentatomophaga latifascia* (Villeneuve) (Diptera: Tachinidae) was found as a parasite attacking overwintering BMSB adults.^10^ Therefore, it is highly possible to develop a biologically‐based integrated pest management (IPM) approach to control overwintering BMSB adults and their eggs laid in the beginning of the season, thereby suppressing BMSB populations during the fruit growing season.

Kiwifruit was damaged by BMSB soon after fruit set (i.e., June) and continued throughout the growing season until harvest (i.e., late September–early October) in organic and conventional orchards. Both incidence and severity of BMSB feeding damage were found overall higher in organic orchard than conventional orchard across all months. However, incidence and severity of feeding damage differed significantly between organic and conventional orchards only in some months in 2019 and 2021. These results indicate that spraying chemical pesticides in the conventional orchard was not good enough to reduce damage caused by BMSB in kiwifruit, even though bifenthrin was applied in 2019 and 2021 (May) which was confirmed as the most effective pesticide against BMSB.^33^, ^34^ Therefore, a second bifenthrin application could be applied in conventional orchards in mid‐ to late‐July, before the peaking of first generation BMSB adults, to reduce damage incidence by reducing numbers of ovipositing females that will produce the second generation. In addition, BMSB can move quickly between host plants, either wild or cultivated, and this dispersal behaviour allows them to escape from insecticide applications and recover in untreated areas.^35^, ^36^ Using an action threshold to trigger insecticide applications would be a practical way for BMSB management. Significantly less BMSB fruit damage has been observed in apples when trees were treated weekly or using a threshold to trigger insecticide applications.^19^ Therefore, it would be helpful to further develop an action threshold based on pheromone trap captures in combination with BMSB feeding damage to trigger insecticide applications to effectively manage BMSB on kiwifruit orchards.

The present study lays the foundation for further understanding of BMSB occurrence and impacts on kiwifruit in organic and conventional orchards, and also for the development of ecology‐based IPM approaches against this destructive insect pest.

## AUTHOR CONTRIBUTIONS

J‐PZ, GAA, S‐SS and FZ conceived and designed the experiments. J‐PZ, J‐HC, Z‐DL, X‐YT and W‐JL conducted experiments. MA and GAA analysed data. J‐PZ, GAA and FZ wrote the article. All authors contributed to writing and editing of the article.

## FUNDING INFORMATION

This research was funded by Zespri Group Limited (no. BS1913), Ministry of Agriculture and Rural Affairs of China, Yunnan Province Science and Technology Department‐Yunnan International Joint Laboratory of Fruit‐Vegetable‐Flower Invasive Insect Pest Management (Yunnan FVF‐IPM Joint Lab) (no. 202303AP140018) and China's donation to CABI Development Fund (VM10051).

## CONFLICT OF INTEREST STATEMENT

The authors declare no conflict of interest.

## Data Availability

The data that support the findings of this study are available on request from the corresponding author. The data are not publicly available due to privacy or ethical restrictions.
